# Lead-Free Ternary Glass for Radiation Protection: Composition and Performance Evaluation for Solar Cell Coverage

**DOI:** 10.3390/ma16083036

**Published:** 2023-04-12

**Authors:** Mohamed A. M. Uosif, Shams A. M. Issa, Antoaneta Ene, Ahmed M. A. Mostafa, Ali Atta, Emam F. El Agammy, Hesham M. H. Zakaly

**Affiliations:** 1 Physics Department, College of Science, Jouf University, Sakaka P.O. Box 2014, Saudi Arabia; 2 Physics Department, Faculty of Science, Al-Azhar University, Assiut 71452, Egypt; 3 Department of Physics, Faculty of Science, University of Tabuk, Tabuk 47512, Saudi Arabia; 4 INPOLDE Research Center, Department of Chemistry, Physics and Environment, Faculty of Sciences and Environment, Dunarea de Jos University of Galati, 47 Domneasca Street, 800008 Galati, Romania; 5 Institute of Physics and Technology, Ural Federal University, 620075 Yekaterinburg, Russia

**Keywords:** gamma shielding, Bi_2_O_3_, neutrons, structure, phospho-bismuth glass

## Abstract

Solar cells in superstrate arrangement need a protective cover glass as one of its main components. The effectiveness of these cells is determined by the cover glass’s low weight, radiation resistance, optical clarity, and structural integrity. Damage to the cell covers brought on by exposure to UV irradiation and energetic radiation is thought to be the root cause of the ongoing issue of a reduction in the amount of electricity that can be generated by solar panels installed on spacecraft. Lead-free glasses made of xBi_2_O_3_–(40 − x)CaO-60P_2_O_5_ (x = 5, 10, 15, 20, 25, and 30 mol%) were created using the usual approach of melting at a high temperature. The amorphous nature of the glass samples was confirmed using X-ray diffraction. At energies of 81, 238, 356, 662, 911, 1173, 1332, and 2614 keV, the impact of various chemical compositions on gamma shielding in a phospho-bismuth glass structure was measured. The evaluation of gamma shielding revealed that the results of the mass attenuation coefficient of glasses increase as the Bi_2_O_3_ content increases but decrease as the photon energy increases. As a result of the study conducted on the radiation-deflecting properties of ternary glass, a lead-free low-melting phosphate glass that exhibited outstanding overall performance was developed, and the optimal composition of a glass sample was identified. The 60P_2_O_5_–30Bi_2_O_3_–10CaO glass combination is a viable option for use in radiation shielding that does not include lead.

## 1. Introduction

Not only can radiation sources that are derived through electrical discharge machinery or radioisotopes pose a risk to human health, but they also pose a risk to sensitive laboratory equipment. Because of this, a shielding material of the highest possible quality is necessary in order to reduce the radiation to safe and acceptable levels [1]. Radiation shielding has traditionally been accomplished using materials, such as tiles, concrete blocks, and clay bricks. The utilization of transparent materials, such as glass for shielding purposes, holds great importance, particularly in hot cells, containers for radiation sources, and windows used in medical X-ray imaging facilities. Nonetheless, it remains highly significant. Glass is an example of a material that is transparent [2,3,4].

Glass based on TeO_2_, B_2_O_3_, SiO_2_, and P_2_O_5_ have all been developed by scientists and researchers over the last several decades to make high-quality radiation shielding glass. Other forms of glass based on phosphate and tellurite have also been developed. Most glasses have Pb added to them because lead has a large mass and can produce glass with a high density. Prior research found that incorporating various quantities of lead oxide into borate glass resulted in the production of high-density glass with a volume of up to 5 g cm^−3^, which was then shown to be an effective radiation shielding material [5,6].

Unfortunately, lead exposure may have several negative consequences, including damage to the kidneys and the brain, disturbance of the neurological system, and spontaneous abortions in pregnant women. Both the IARC and the DHHS have concluded that Pb and Pb-compounds are responsible for making the environment hazardous and are probably carcinogenic to people. Bismuth is an essential component in the creation of a radiation shielding material that can successfully replace lead. As compared to lead-silicate-based glass, silicate-based glass that has had bismuth oxide (Bi_2_O_3_) added to it produces a material with a greater density and demonstrates improved shielding capabilities [7,8].

The history of research on phosphate glass is extensive. In contrast to borosilicate glass, the glass in question has a lower temperature at which it melts. Phosphate glass, by contrast, has a chemical resistance that is not very high. Several researchers have found that by adding CaO in phosphate glass, they are able to increase the chemical endurance of the material. According to several studies, divalent ions, such as Ca^2+^, are capable of forming a P–O–Ca covalent bond as well as acting as an ionic cross-linking agent between the NBO groups of two different cross-linked chains, which results in an increase in the chemical durability of the material [9,10]. The incorporation of glass into the composition will be facilitated by the addition of an alkaline component, such as CaO, which will also decrease the glass’s melting temperature, lessen its propensity to crystallize, and result in improvements to the glass’s other physical and chemical characteristics. When there is an excessive amount of CaO in the glass, the brittleness of the glass will readily rise [11,12].

Many academics have assessed the comparison results for the shielding parameter between the WinXcom software and experimental data. The calculation for the shielding parameter may be used for polymer [13], concrete [14], alloy [15], and compound [16] in addition to glasses. There is not a limitation on its applicability. The shielding parameter values that were provided from their works were analyzed by the WinXCOM software, and the results showed that they were compatible with the experimental value, within the experimental errors.

Yin Zhang and colleagues conducted research on the P_2_O_5_–Bi_2_O_3_–CaO system to determine the glass forming area, structure, and features of the system [17]. Y. Chaitanya conducted research on the impact that copper ions have on the physicochemical, structural, spectroscopic, and dielectric characteristics of Bi_2_O_3_–CaO–P_2_O_5_–B_2_O_3_ glasses [18]. Zainab Mufarreh Elqahtani et al. showed that Bi_2_O_3_ has an impact on the optical qualities as well as the radiation attenuation characteristics of BiO_3_–Li_2_O–P_2_O_5_ glasses [19]. Producing and describing glassy waste forms based on the SrF_2_–Fe_2_O_3_–PbO/BiO_3_–P_2_O_5_ system was conducted by Xiuying Li et al. [20]. W. Rachniyom and colleagues investigated how the presence of Bi_2_O_3_ changes the radiation-blocking capabilities of glasses made from coal fly ash [21]. The originality of the work resides in the efficiency of Bi_2_O_3_–CaO–P_2_O_5_ system against radiation at 81, 238, 356, 662, 911, 1173, 1332, and 2614 keV. In addition, there is no study focused on the capability of this system to absorb gamma radiation.

The purpose of the current study is to carry out the investigation of lead-free glass systems that contain bismuth oxide and to investigate the possibilities of these systems becoming new candidates for a gamma-shielding material, particularly in observation windows in radiotherapy rooms and control rooms of nuclear power plants. The effects of bismuth oxide in a phospho-bismuth glass system will be reported in this research. The focus of this study will be on two features: (1) the physical and structural properties, and (2) the gamma-radiation-shielding capabilities. Because of its one-of-a-kind qualities, such as excellent transparency, strong gamma-shielding capabilities, and great heat resistance when combined with heavy metal oxides, phospho-bismuth was selected as the basis glass for this investigation. It is anticipated that the incorporation of bismuth oxide into phospho-bismuth glass would result in an increase in the density of the glass as well as an improvement in its capacity to shield gamma radiation.

## 2. Experimental Procedure

The usual method of melt quenching was used for the production of xBi_2_O_3_–(40 − x)CaO-60P_2_O_5_ (where x = 5, 10, 15, 20, 25, and 30 mol%). The high-purity raw ingredients NH_4_H_2_PO_4_, Bi_2_O_3_, and CaO were each precisely weighed at 10 g per batch before being ground into a powder and uniformly mixed. The combined components are then transferred to a crucible made of alumina. After that, it was melted in an electrical furnace, and the temperature of the furnace was raised to around 420 degrees Celsius at a pace of 5 degrees Celsius per minute, where it remained for an hour. After being poured into the heated mold, the totally molten liquid was annealed at 440 degrees Celsius for one hour, allowed to cool to ambient temperature, and then removed from the form. To evaluate the gamma-shielding capabilities of glass, a portion of the produced glass samples were shaped into cylinders of varying thicknesses after being cut, ground, and polished. In preparation for the XRD analysis, the remaining glass samples are crushed into powder [17]. Table 1 presents the design components that are found in glass [17].

**Figure 1:**
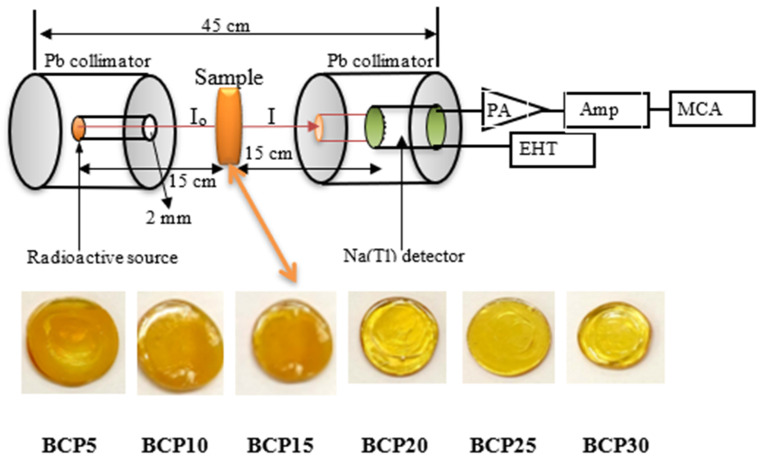
Experimental radiation measurements setup and fabricated samples of xBi_2_O_3_–(40 − x)CaO-60P_2_O_5_ glasses in mol%.

To determine that the sample material in question was amorphous, we used a Shimadzu XD-DI X-ray diffractometer operating under a set of working circumstances consisting of 40 kV and 30 mA for each sample. The density (ρ) of the glass sample that has been manufactured is determined by submerging it in water that has been distilled in accordance with the principle of Archimedes. Using the narrow beam method with a lead-collimator to experimentally estimate linear attenuation coefficients (G_LAC_), values were acquired using a multichannel analyzer coupled to a NaI (Tl)-scintillation detector with a 3 × 3-inch measurement area. ^133^Ba (80 and 356 keV), ^137^Cs (662 keV), ^60^Co (1173 and 1333 keV), and ^232^Th (238, 911 and 2614 keV) have all been employed in the experiment. Source, sample, and detector locations are shown in their experimental configurations in Figure 1 [22,23]. For each gamma line, the photon intensity was calculated without and with an absorber by using the area under the photopeak. Five repetitions of the process were carried out, each lasting 10 min. The margin of error was less than one percent.

## 3. Results and Discussions

Damage to the cell covers brought on by exposure to UV irradiation and energetic radiation is thought to be the root cause of the ongoing issue of a reduction in the amount of electricity that can be generated by solar panels installed on spacecraft. A shorter lifespan places restrictions on the space mission and necessitates replacement at a very expensive cost. The cover glasses have the dual purpose of enhancing transmission while also blocking harmful radiation. There is a lack of understanding on the causes of radiation-induced transmission loss. To manufacture cover glasses with greater immunity to contamination and lower defect densities than those produced by the current coating techniques, we intentionally produced glasses with these characteristics. Enhancements will be made to analytical procedures that have been established for the purpose of assessing the characteristics of glass and linking those attributes to radiation tolerance. Our experience with emerging technologies and the impacts of the radiation environment in space on optics are centered on this topic, and we believe that it will contribute to a better understanding of the processes that are responsible for the darkening caused by radiation. An increase in the radiation resistance of the optical glass that was placed on the cover glasses would have the effect of extending the end of life for applications both in the civilian and military spheres. This would be beneficial. The reduction in our reliance on outside sources would be facilitated by the establishment of a local provider of glass cell coverings.

### 3.1. Structural Properties

**Figure 2:**
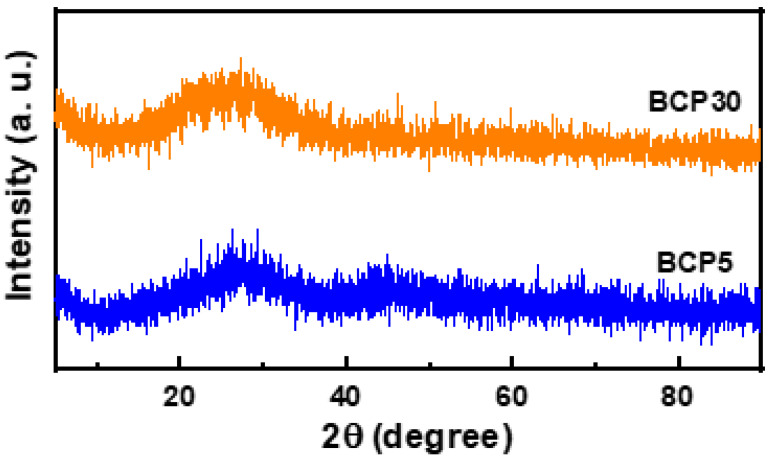
XRD pattern for prepared glasses.

X-ray diffraction patterns for BCP5 and BCP30 glasses are shown in Figure 2, and the patterns are almost identical across all glasses. This diagram does not depict a single, distinct peak in the spectrum, but rather a diffuse ring centered around 25–35°. The pattern in all the glass samples is almost identical to that in Figure 2. All the glass samples used in this study were found to be amorphous, as predicted by the lack of a sharp peak, which implies the absence of a long-range order in the atomic arrangements.

### 3.2. Physical Properties

Glass density may be understood in terms of the degree of variation in the many structural units that make up the glass’s composition. In most cases, the presence of heavy metal oxide in glass results in a denser product. There are six distinct densities of glasses that may be made by adjusting the quantity of Bi_2_O_3_ used: 2.212, 2.609, 3.007, 3.404, 3.802, and 4.199 g/cm^3^. As the percentage of Bi_2_O_3_ in the glass rises, so does the density of the finished product. The glass modifier Bi_2_O_3_ (465.96 g mol^−1^) has a higher molecular weight than CaO (56.0774 g mol^−1^), and this is the reason why the density of glass increased. In addition, by exchanging the low-density oxide CaO (3.34 g cm^−3^) with the high-density oxide Bi_2_O_3_ (8.99 g cm^−3^), the density of the existing glass system is increased. The rise in the proportion of nonbridging oxygen atoms is likely to blame for the increased density of glasses. In addition, the presence of modifier ions, such as Bi^3+^, will try to fill up the voids in the network and ultimately lead to a denser glass. Furthermore, the oxide network loosens up when the Bi_2_O_3_ concentration rises, resulting in a drop in the oxygen packing density of silicate-based glasses [24].

### 3.3. Shielding Parameter

#### 3.3.1. Linear and Mass Attenuation Coefficient

**Figure 3:**
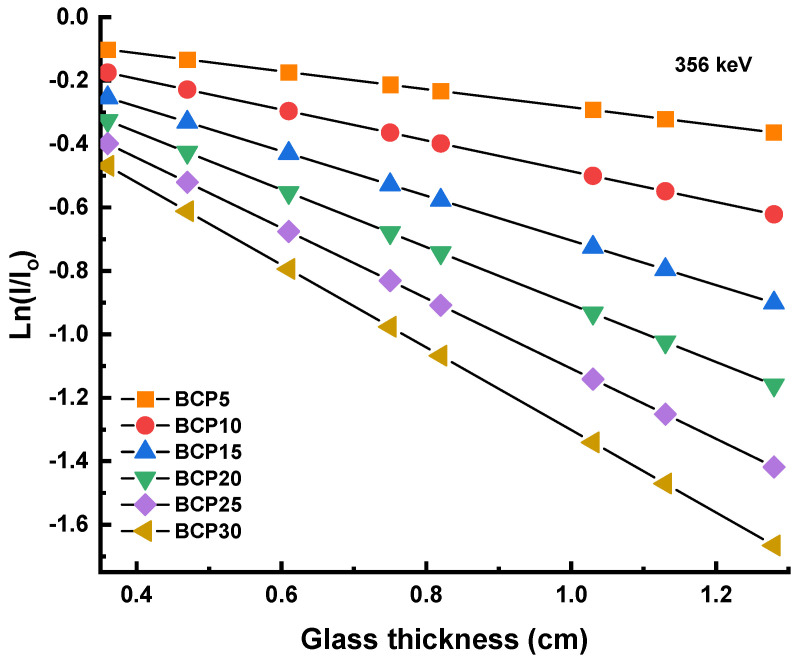
Graph of Ln(I/I_o_) against glass thickness at 356 keV.

Gamma rays from ^133^Ba, ^137^Cs, ^60^Co, and ^232^Th are being used for the measuring process in the narrow beam geometry approach. The linear attenuation coefficient (G_LAC_) was determined by measuring the incident (I_o_) and transmitted (I) gamma-ray intensities, and the resulting value was then utilized to obtain the mass attenuation coefficient (G_MAC_). The linear attenuation coefficient values are obtained by calculating the slope of the linear graph Ln(I/I_o_) against the sample thickness. Ln(I/I_o_) against the sample thickness for all glasses at 356 keV is shown in Figure 3, and the patterns are almost identical across all photon energies. On average, as shown in Figure 3, the slope of the graph rises when more Bi_2_O_3_ is added. The slope of the graph is increased from 1.2479 to 5.7205 cm^−1^ at 81 keV, from 0.4247 to 1.9469 cm^−1^ at 238 keV, from 0.2839 to 1.3016 cm^−1^ at 356 keV, from 0.1527 to 0.6999 cm^−1^ at 662 keV, from 0.1110 to 0.5086 cm^−1^ at 911 keV, from 0.0862 to 0.3950 cm^−1^ at 1173 keV, from 0.0759 to 0.3479 cm^−1^ at 1333 keV, and from 0.0387 to 0.1773 cm^−1^ at 2614 keV when the Bi_2_O_3_ content increased from 5 to 30 mol%. In this particular series of glasses, the glass with the greatest concentration of Bi_2_O_3_ had the largest gradient, which may be translated as the highest values of G_LAC_ in comparison to other glasses. Take note that the Z of Bi is 83, which is much higher than the number for Ca (20).

**Figure 4:**
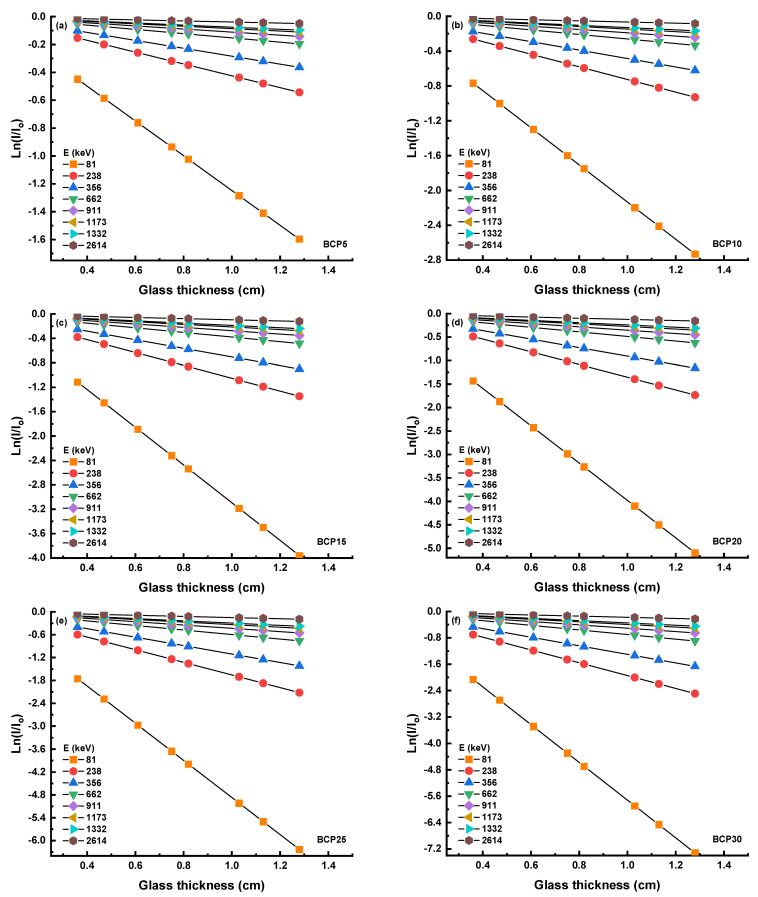
(**a**–**f**) Graph of Ln(I/I_o_) against glass thickness at selected photon energy.

The interaction between gamma ray photons and the atoms of Bi will become more intense if a large atomic number of Bi is added to the structure of the glass. The higher the atomic number, the greater the amount of photon energy that must be absorbed before an electron may be ejected from a Bi atom. Either the photoelectric effect or the Compton scattering might have been responsible for the expelled electron. When there is a greater amount of interaction between gamma rays and the target atom (Bi), the number of rays that are transmitted through the glass is reduced. The G_LAC_ rose as a direct result of this reason [25]. The ρ of the glass modifier is still another element that contributed to the rise in the value of the G_LAC_. It is thought that decreasing the porosity nature of the glass and producing high ρ glass may be accomplished by introducing a high ρ modifier (Bi_2_O_3_ = 8.9 g m^−3^) into the glass system. Since glass has a lesser porosity than other materials, it will have a greater attenuation than other materials because there is a greater possibility that gamma rays will interact with the atoms in glass [26]. Figure 4a–f shows the Ln(I/I_o_) as the function of glass thickness for BCP5, BCP10, BCP15, BCP20, BCP25, and BCP30 samples at 81, 238, 356, 662, 911, 1173, 1332, and 2614 keV. As seen in these figures the slopes decrease as the energy increased. It means that the G_LAC_ values for certain glass decrease with increasing energy. The results found that the highest G_LAC_ value was at 81 keV while the lowest was at 2616 keV.

**Figure 5:**
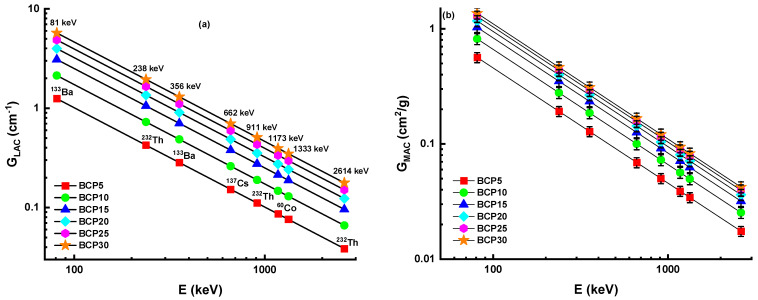
(**a**) Linear attenuation coefficient (G_LAC_) and (**b**) mass attenuation coefficient (G_MAC_) for glass samples.

Figure 5a,b shows the G_LAC_ and G_MAC_ values for BCP5, BCP10, BCP15, BCP20, BCP25, and BCP30 samples at 81, 238, 356, 662, 911, 1173, 1332, and 2614 keV, respectively. If you look at the graph in Figure 5a,b, you will see that the G_LAC_ and G_MAC_ values rise when the concentration of Bi2O3 rises. This is something that can be observed. The xBi_2_O_3_–(40 − x)CaO-60P_2_O_5_ glass system has the greatest G_LAC_ and G_MAC_ values, which are, respectively, 5.7205 cm^−1^ and 1.3624 cm^2^/g. The maximal concentration of Bi_2_O_3_ in the glass system contributed to the highest G_LAC_ and G_MAC_, as was predicted. The more the G_LAC_ and G_MAC_ values are, the better a certain material is in attenuating a greater number of photons. At the energies that were investigated, it was found that increasing the amount of Bi_2_O_3_ in the glass samples led to an increase in both the G_LAC_ and G_MAC_. The reason for this is that the presence of Bi_2_O_3_ raises their effective atomic numbers as well as their densities. As a result of the fact that BCP30 glass (Bi_2_O_3_ = 30 mol%) exhibited the greatest density when compared to other glasses, it provided the highest G_MAC_ value. This revealed that the BCP30 glass had the maximum photon interaction at the given energy level. This interaction may take place as a result of the photoelectric effect (PE), Compton scattering (CS), or pair production (PP). In general, there are four different scenarios in which gamma ray photons might cause glass material to become irradiated: (a) Photons pass through the glass without causing any interaction; (b) photons are absorbed directly into the atoms that make up the structure of the glass through PE; (c) photons interact with the glass through CS and pass through the glass; and (d) atoms interact with photons through CS multiple times before being absorbed by PE [27].

#### 3.3.2. Half-Value Layer and Mean-Free Path

**Figure 6:**
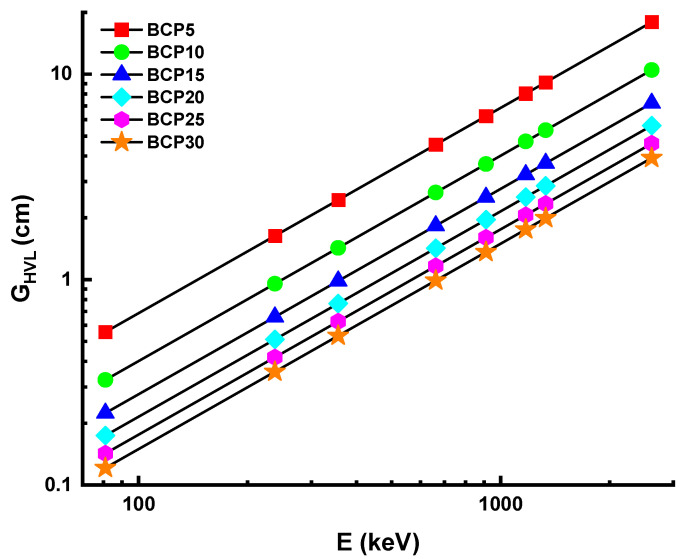
Variation of half-value layer (G_HVL_) for prepared glasses.

**Figure 7:**
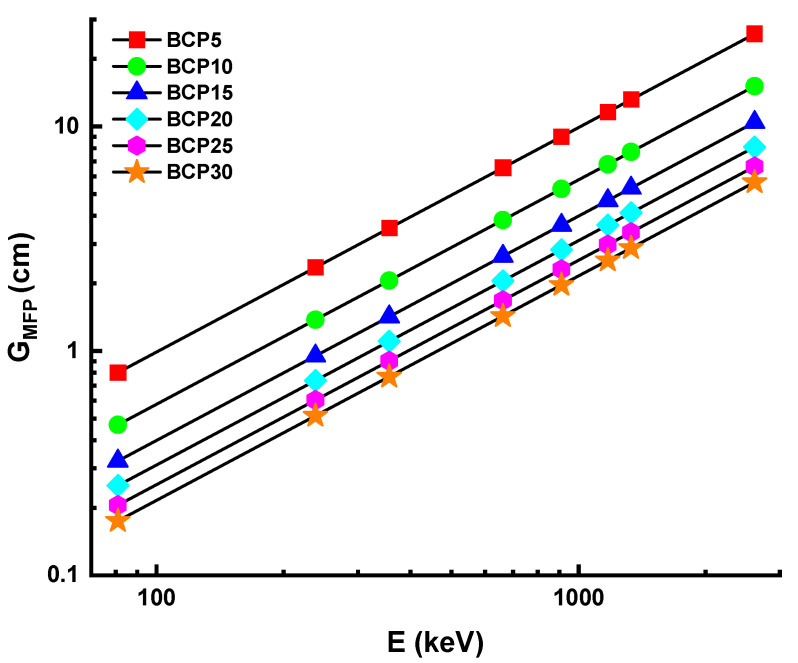
Variation of mean-free path (G_MFP_) for prepared glasses.

It is also possible to describe the efficiency of gamma shielding in terms of the half-value layer (G_HVL_) and the mean-free path (G_MFP_). Better shielding material may be produced using material that has a lower value of both the G_HVL_ and G_MFP_. The thickness of the material that is required to absorb fifty percent of the incoming radiation is referred to as the G_HVL_, and the G_MFP_ is the average distance traveled by the photon between two subsequent contacts. Figure 6 and Figure 7 each provide a scatter plot depicting the values of the G_HVL_ and G_MFP_ for each of the glasses. With the addition of Bi_2_O_3_, there is a discernible shift in both the G_HVL_ and G_MFP_ values of glasses. When the mol percentage of Bi_2_O_3_ grew to its maximum quantity, both the G_HVL_ and G_MFP_ value fell. The increasing pattern of G_HVL_ and G_MFP_ values for glasses may be ascribed to the increase in density and the G_MAC_ of glasses. This pattern shows a decreasing value for each parameter. In the present study, the G_HVL_ and G_MFP_ values of BCP30 glass are found to be the lowest among those of other glasses. A total of 0.12, 0.36, 0.53, 0.99, 1.36, 1.75, 1.99, and 3.91 cm are the G_HVL_ values for the BCP30 sample at 81, 238, 356, 662, 911, 1173, 1332, and 2614 keV, respectively. A total of 0.17, 0.5, 0.77, 1.43, 1.97, 2.53, 2.87, and 5.64 cm are the G_MFP_ values for the BCP30 sample at 81, 238, 356, 662, 911, 1173, 1332, and 2614 keV, respectively. This demonstrates that BCP30 glass is superior to other types of shielding glass in terms of its ability to reduce the number of photons produced by gamma rays and its overall effectiveness.

**Figure 8:**
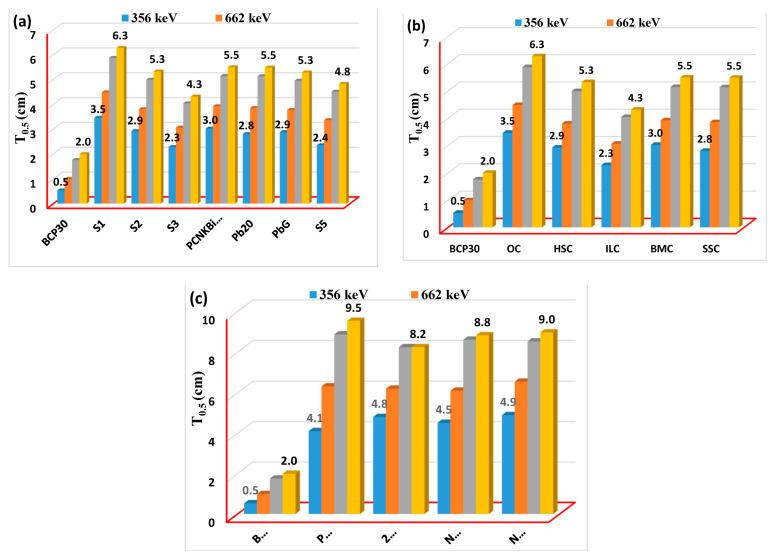
Half-value layer values of a BCP30 glass sample compared to (**a**) glass materials, (**b**) some concrete, and (**c**) polymers at 356, 662, 1173, and 1333 keV.

In light of the fact that the BCP30 has the lowest G_HVL_ value among the glasses that were investigated, it is compared with certain common gamma-shielding glasses, concretes, and polymers as shown in Figure 8a–c at 356, 662, 1173, and 1333 keV. In this figure, the G_HVL_ value of the BCP30 glass is lower than the G_HVL_ values of the different types of glass materials, concretes, and polymers. It means that this glass sample better absorbs than S1 [28], S2 [29], S3 [30], PCNKBi7.5 [31], Pb20 [7], PbG [32], S5 [33], (OC, HSC, ILC, BMC, IC) concretes [34], and PbCl2(20%) [35], 20% BaZrO3 [36], NPW20 [37], and Nb(15%) [38]. At a selected photon energy, it has been shown that the glass produced by the current research is more effective than some glasses, concretes, and polymers. Considering these findings, it is possible to draw the conclusion that BCP30 may be a viable option for use as a radiation-shielding material.

#### 3.3.3. Radiation Protection Efficiency

**Figure 9:**
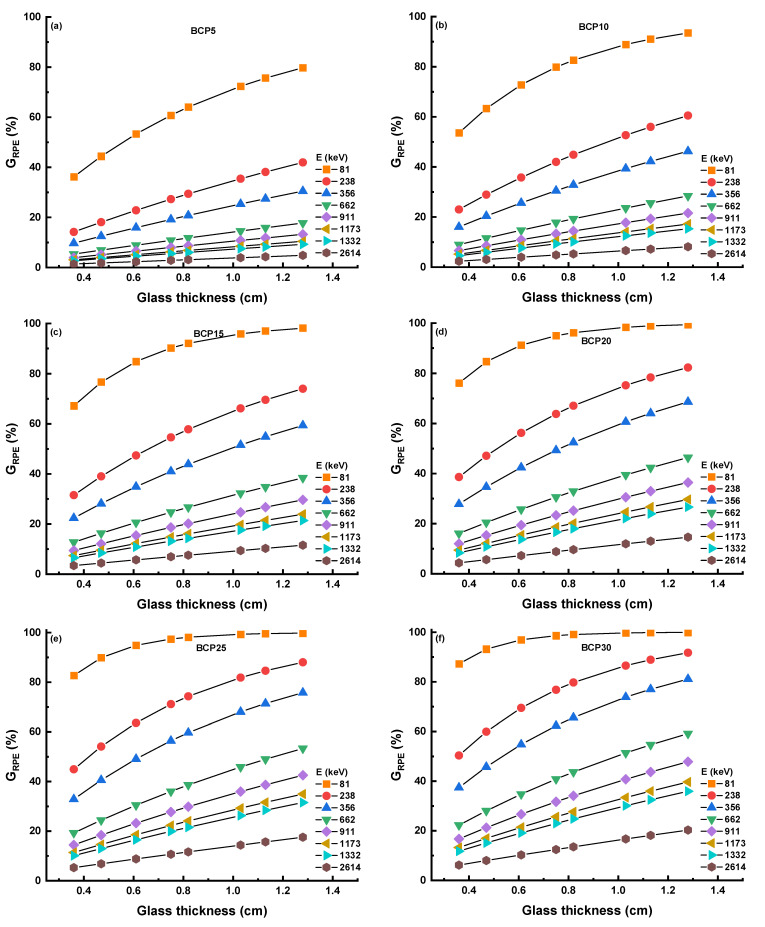
(**a**–**f**) Gamma radiation protection efficiency (G_RPE_) for all glasses at selected photon energy.

**Figure 10:**
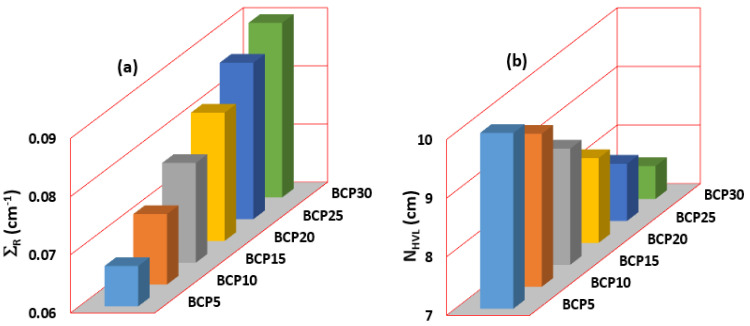
(**a**) Fast neutron removal cross-sections (Σ_R_) and (**b**) half-value layer for neutron radiation N_HVL_ values for all glass samples.

Gamma radiation protection efficiency (G_RPE_) is the other important factor that indicates how the glass is efficient in absorbing the photons. Figure 9a–f shows the G_RPE_ values for BCP5, BCP10, BCP15, BCP20, BCP25, and BCP30 samples at 81, 238, 356, 662, 911, 1173, 1332, and 2614 keV, respectively. As seen in this figure, the G_RPE_ values for glasses increase with both the increasing thickness and concentration of glasses and decrease with the increasing photon energy. A total of 99.93, 91.73, 81.10, 59.18, 47.85, 39.69, 35.94, and 20.30% are the G_RPE_ values for BCP30 samples at 81, 238, 356, 662, 911, 1173, 1332, and 2614 keV, respectively. It means that the highest G_RPE_ value at a lower energy and the lowest G_RPE_ value at the highest energy. The effective removal of the cross-section, also known as Σ_R_, has been responsible for the creation of the glass samples. It is possible to make the assertion that there is a linear connection between the amount of Bi_2_O_3_ present in the glass materials and the values of Σ_R_, as shown in Figure 10a. The results of Σ_R_ indicate that the BCP30 sample has the greatest value. The Σ_R_ values have been used to calculate the neutron half-value layer N_HVL_ for all glass samples [39]. Figure 10b shows that the N_HVL_ values decrease as the Bi_2_O_3_ content increases, and the BCP30 sample has the lowest value.

The finding discusses the X-ray diffraction patterns and gamma ray attenuation coefficients for Bi_2_O_3_-CaO-P_2_O_5_ (BCP) glasses. The X-ray diffraction patterns for BCP5 and BCP30 glasses are almost identical, depicting a diffuse ring centered around 25–35°, which indicates that all glass samples used in the study were amorphous. The density of the glass is determined by the degree of variation in the many structural units that make up the glass’s composition, and the presence of heavy metal oxide in glass results in a denser product. Bi_2_O_3_ is a glass modifier with a higher molecular weight than CaO, and the glass density increases with an increase in Bi_2_O_3_ concentration due to the presence of modifier ions, such as Bi^3+^, and a rise in the proportion of non-bridging oxygen atoms. The addition of Bi_2_O_3_ to the glass structure increases the interaction between gamma ray photons and the atoms of Bi, resulting in a higher value of the linear attenuation coefficient (G_LAC_) as the atomic number of Bi is higher than that of Ca. The G_LAC_ also increases with an increase in the density of the glass, and glass with a higher density produces a higher value of G_LAC_. However, the G_LAC_ values for certain glass decrease with increasing energy.

The findings are significant in understanding the properties of BCP glasses, particularly in relation to their density and gamma ray attenuation. The results show that the addition of Bi_2_O_3_ to the glass structure can significantly increase its density, which can have important applications in various fields, such as radiation shielding, where high-density materials are required. This study also highlights the importance of the atomic number of glass modifiers, such as Bi_2_O_3_, in determining the attenuation of gamma rays, which can have implications in fields, such as nuclear medicine, where gamma ray imaging and therapy are commonly used. Furthermore, the finding that the G_LAC_ values for certain glass decrease with increasing energy can have important applications in the design of radiation detectors, where the attenuation of gamma rays at different energies is an important consideration. Finally, the presented finding provides valuable insights into the properties of BCP glasses, particularly in relation to their density and gamma ray attenuation. This study highlights the importance of glass modifiers, such as Bi_2_O_3_, in determining the properties of glass, and the findings can have important applications in various fields, such as radiation shielding and nuclear medicine. However, further research is needed to explore the effect of other factors on the properties of BCP glasses and their applications in different fields.

## 4. Conclusions

In the current investigation, the density of the phospho-bismuth glass system was made denser by the use of a glass modifier, known as Bi_2_O_3_. When compared to different types of glass, it was discovered that the glasses with the greatest Bi_2_O_3_ concentration produced the highest G_MAC_ while simultaneously having the lowest G_HVL_. The X-ray diffraction patterns for BCP5 and BCP30 glasses showed a diffuse ring centered around 25–35°, indicating that all the glass samples used in the study were amorphous. The linear attenuation coefficient (G_LAC_) was determined for all glasses at different photon energies using gamma rays from different sources. The G_LAC_ values increased as the concentration of Bi_2_O_3_ in the glass increased, which was attributed to the interaction between gamma ray photons and the atoms of Bi and the density of the glass modifier. Furthermore, the G_LAC_ values for certain glass decreased with increasing energy. This glass sample produced an outcome that is even superior to regular concretes and some radiation shielding polymers. This suggests that this glass is more effective at reducing the effects of gamma rays and offers a higher level of protection than other glasses. The results concluded that the prepared glass samples can be used as a cover for the solar cells to protect them from harmful radiation. due to the great ability of the prepared glasses to attenuate the radiation, especially the BCP30 sample that has the highest Bi_2_O_3_ content.

## Figures and Tables

**Table 1:** Elemental glass composition in fractional weight.

Elements	BCP5	BCP10	BCP15	BCP20	BCP25	BCP30
Bi	0.163149	0.281292	0.370796	0.440947	0.497411	0.543837
O	0.43717	0.38764	0.350117	0.320707	0.297035	0.277572
Ca	0.10951	0.080919	0.059259	0.042282	0.028618	0.017383
P	0.290171	0.250148	0.219828	0.196063	0.176936	0.161208

## Data Availability

The datasets generated during and/or analyzed during the current study are available from the corresponding author upon reasonable request.
